# Effects of Oxytocin on Fear Memory and Neuroinflammation in a Rodent Model of Posttraumatic Stress Disorder

**DOI:** 10.3390/ijms19123848

**Published:** 2018-12-03

**Authors:** Sheng-Chiang Wang, Chen-Cheng Lin, Chun-Chuan Chen, Nian-Sheng Tzeng, Yia-Ping Liu

**Affiliations:** 1 Graduate Institute of Medical Sciences, National Defense Medical Center, Taipei 11490, Taiwan; abnerwang@hotmail.com; 2 Department of Psychiatry, Tri-Service General Hospital Songshan Branch, Taipei 10581, Taiwan; 3 Department of Psychiatry, Cheng Hsin General Hospital, Taipei 11220, Taiwan; wsadhjkl@gmail.com; 4 Laboratory of Cognitive Neuroscience, Department of Physiology and Biophysics, National Defense Medical Center, Taipei 11490, Taiwan; jim91036@yahoo.com.tw; 5 Department of Psychiatry, Tri-Service General Hospital, Taipei 11490, Taiwan; pierrens@mail.ndmctsgh.edu.tw; 6 Student Counseling Center, National Defense Medical Center, Taipei 11490, Taiwan

**Keywords:** posttraumatic stress disorder, single prolonged stress, oxytocin, pro-inflammatory cytokines, tumor necrosis factor α, interleukin 1β, interferon γ

## Abstract

Posttraumatic stress disorder (PTSD) is a trauma-induced mental disorder characterized by fear extinction abnormalities, which involve biological dysfunctions among fear circuit areas in the brain. Oxytocin (OXT) is a neuropeptide that regulates sexual reproduction and social interaction and has recently earned specific attention due to its role in adjusting neurobiological and behavioral correlates of PTSD; however, the mechanism by which this is achieved remains unclear. The present study aimed to examine whether the effects of OXT on traumatic stress-induced abnormalities of fear extinction (specifically induced by single prolonged stress (SPS), an animal model of PTSD) are associated with pro-inflammatory cytokines. Seven days after SPS, rats received intranasal OXT 40 min before a cue-dependent Pavlovian fear conditioning-extinction test in which rats’ freezing degree was used to reflect the outcome of fear extinction. We also measured mRNA expression of *IL-1β*, *IFN-γ*, and *TNF-α* in the medial prefrontal cortex (mPFC), hippocampus, and amygdala at the end of the study, together with plasma oxytocin, corticosterone, IL-1β, IFN-γ, and TNF-α, to reflect the central and peripheral changes of stress-related hormones and cytokines after SPS. Our results suggested that intranasal OXT effectively amends the SPS-impaired behavior of fear extinction retrieval. Moreover, it neurochemically reverses the SPS increase in pro-inflammatory cytokines; thus, *IL-1β* and *IFN-γ* can be further blocked by the OXT antagonist atosiban (ASB) in the hippocampus. Peripheral profiles revealed a similar response pattern to SPS of OXT and corticosterone (CORT), and the SPS-induced increase in plasma levels of IL-1β and TNF-α could be reduced by OXT. The present study suggests potential therapeutic effects of OXT in both behavioral and neuroinflammatory profiles of PTSD.

## 1. Introduction

Posttraumatic stress disorder (PTSD) is a mental disorder characterized by fear memory abnormalities in individuals who have previously experienced a traumatic event [1,2]. The cardinal psychopathology of PTSD is an overactive fright response which reflects a failure of the fear extinction process in objectively safe situations [3]. In addition, clinical findings have shown that PTSD patients usually become unable to forget the trauma-associated memory [4,5,6]. The underlying mechanism of PTSD remains unsolved, yet it should primarily involve the excessive feedback of the hypothalamus (HT)–pituitary–adrenal (HPA) axis [1,7] as well as a biological imbalance among the fear circuit areas in the brain, namely the medial prefrontal cortex (mPFC), hippocampus, and amygdala [7,8,9]. With such complicated pathological attributes, treatment of PTSD is difficult and truly effective treatments have not yet been reported [10].

Oxytocin (OXT) is a hypothalamic neuropeptide which plays not only a hormonal role in sexual reproduction, but also acts as a neuropeptide in the brain to regulate face recognition and social interaction [11,12]. Increasing evidence suggests that oxytocin may have some potential role in adjusting neurobiological and behavioral correlates of PTSD [13,14]. A human image study suggested that this is possibly because oxytocin modulates impairments associated with cognitive deficits in domains such as working memory and executive control—which are functions executed via the prefrontal cortex, the impairment of which is highly prevalent among individuals with PTSD [13]. A rodent study also supported the utility of oxytocin by demonstrating that prophylactic intranasal oxytocin administered concurrently with aversive stimuli can alleviate traumatic stress-induced behavioral and physiological responses [15]. Taken together, oxytocin may exert some effects on PTSD-associated behaviors in connection with abnormalities of fear circuit areas, yet the underlying mechanism by which this is achieved remains unclear.

Cumulative evidence suggests that neuroinflammation may be a major contributor in the pathoetiology of many psychiatric disorders [16], including oxytocin-involved stress-related mental illness. Oxytocin may exert anti-inflammatory properties to mitigate maternal separation stress-induced depressive behaviors [17]. In fact, oxytocin is implicated in the stress-associated pathophysiology, particularly in terms of central–peripheral stress regulation, as it is greatly associated with HPA dysfunction observed following traumatic stress. For example, central OXT has been shown to modulate the cognitive effects of stress [18]. OXT inhibits the action of corticotropin-releasing hormone (CRH), which induces the release of cortisol during stress response [19]. It is possible that central OXT signaling exerts a buffering effect against stressful events and experiences, thereby reducing vulnerability to psychopathology and physical illness [20].

As a stress-related mental disorder, PTSD is of interest in terms of its pathological attributes related to immune system dysregulation by reason of the following: first, immune function and stress hormones can mutually affect PTSD vulnerability and predictability [21,22,23,24]; second, the release of inflammatory cytokines, as an immune response, can influence stress vulnerability via neural circuitry [24]; and third, pro- and anti-inflammatory cytokines operate in fear circuit areas in the brain such as the prefrontal cortex (PFC), hippocampus, and amygdala [25,26].

The present study aims to clarify whether the therapeutic effect of oxytocin on fear memory abnormalities is associated, or mediated, with changes of central and peripheral immune functions. We examined both behavioral and biochemical changes under the pharmacological interventions of OXT and OXT antagonist in rats that were previously exposed to traumatic stress. Single prolonged stress (SPS), modified from Liberzon and colleagues [27,28], was employed to model PTSD. SPS has been recognized to simulate a traumatic stressful event and, when employed together with a cue-dependent Pavlovian fear conditioning-extinction test (CDFC) [29,30,31], as was done in the present study, its yields high validity levels in examining the core psychopathology of PTSD, i.e., trauma-disrupted cue/contextual extinction learning [32,33]. In the present study, we also measured central interleukin-1β (*IL-1β*), interferon-γ (*IFN-γ*), and tumor necrosis factor-α (*TNF-α*) in the medial prefrontal cortex (mPFC), hippocampus, and amygdala, together with plasma oxytocin, corticosterone, IL-1β, IFN-γ, and TNF-α, to investigate the central and peripheral changes of stress-related hormones and cytokines after SPS. The results of the present study not only are helpful in providing valuable information of the clinical potential of oxytocin in treating PTSD, but also useful in investigating the role of neuroinflammation in the pathoetiology of PTSD.

## 2. Results

### 2.1. OXT (Oxytocin) Mitigated SPS-Induced Impairment of Fear Extinction

**Figure 1:**
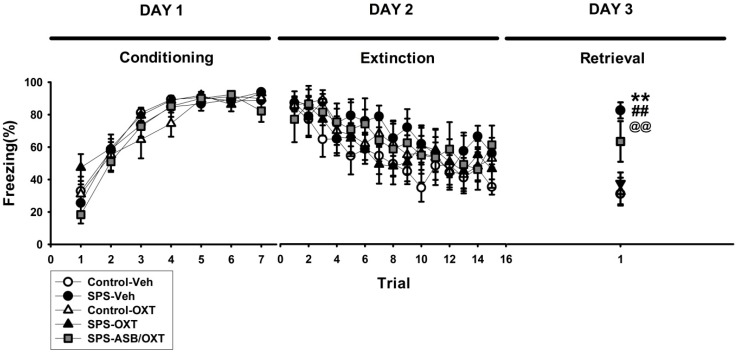
Cue-dependent fear conditioning–extinction test (CDFC test). Oxytocin (OXT) reversed SPS-attenuated fear extinction retrieval (more freezing level in the DAY 3 retrieval stage) and the OXT-related reversal phenomena seemed to be blocked by atosiban (ASB). *n* = 8 for Control-Veh, SPS-Veh, Control-OXT, and SPS-OXT; *n* = 6 for SPS-ASB/OXT. Bars represent mean ± SEM. ** *p* < 0.01, control-Veh vs. SPS-Veh; ^##^ *p* < 0.01, SPS-Veh vs. control-OXT; ^@@^ *p* < 0.01, SPS-Veh vs. SPS-OXT. SPS: single prolonged stress; Veh: vehicle, OXT: oxytocin, ASB: atosiban.

We subjected the rats to the CDFC test to determine whether OXT treatment mitigated SPS-induced fear-related behavioral impairments. For the percentage of freezing level in the conditioning stage (Day 1 of CDFC test), there was a significant main effect of TRIAL (F_(6,198)_ = 119.32, *p* < 0.001), but no others main effects or interactions were found (Figure 1, left panel). For the extinction stage (Day 2 of CDFC test), the data revealed a significant main effect of TRIAL (F_(14,462)_ = 11.653, *p* < 0.001), but no others main effects or interactions were found (Figure 1, middle panel). For the percentage of freezing level in the extinction retrieval stage (Day 3 of CDFC test), ANOVA revealed a significant difference among all groups (F_(4,33)_ = 8.83, *p* < 0.001), further analysis exhibited that SPS-Veh group exhibited more freezing level (i.e., impaired extinction retrieval) than the control-Veh (*p* = 0.001), control-OXT (*p* = 0.002), and SPS-OXT groups did (*p* = 0.005) (Figure 1, right panel).

### 2.2. Intranasal OXT Eliminated SPS-Induced Reduction of Peripheral CORT and OXT Levels.

**Figure 2:**
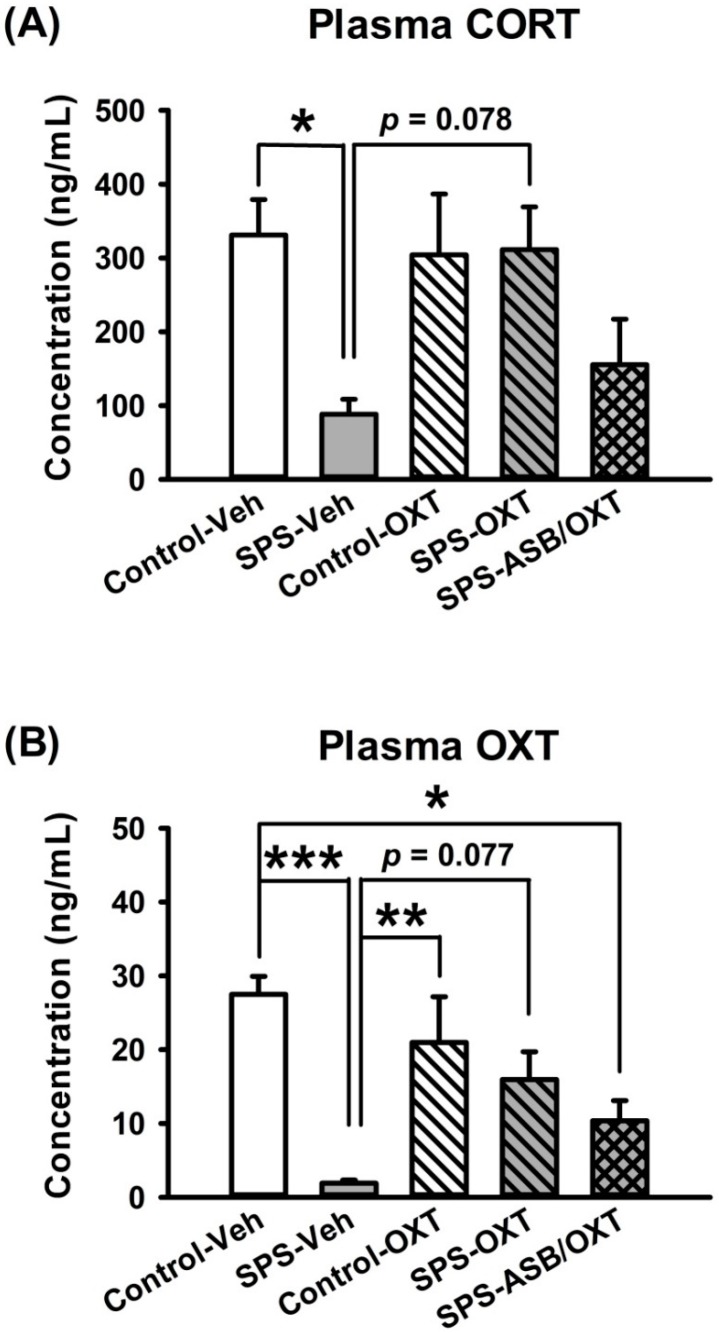
Plasma levels of corticosterone (CORT) and OXT. OXT reversed the SPS (single prolonged stress)-induced reduction of plasma CORT and OXT levels, but ASB blocked it. *n* = 6 for each group, SPS: single prolonged stress; Veh: vehicle; OXT: oxytocin; ASB: atosiban. Bars represent mean ± SEM; * *p* < 0.05, ** *p* < 0.01, *** *p* < 0.001.

Plasma CORT level revealed a significant difference among all groups (F_(4,25)_ = 3.565, *p* = 0.02), deriving from lower CORT level in the SPS rats compared with the control-Veh (*p* = 0.047) and SPS-OXT groups (*p* = 0.078) (Figure 2A). For the plasma OXT level, one-way ANOVA revealed a significant difference among all groups (F_(4,25)_ = 7.286, *p* < 0.001), further analysis exhibited that the plasma OXT level in SPS-Veh group was lower than control-Veh (*p* < 0.001), control-OXT (*p* = 0.008), and SPS-OXT groups (*p* = 0.077), SPS-ASB/OXT group also showed a lower plasma OXT level compared with control-Veh group (*p* = 0.021) (Figure 2B).

### 2.3. Dynamic Region-Dependent Changes of Central Pro-Inflammatory Cytokines

**Figure 3:**
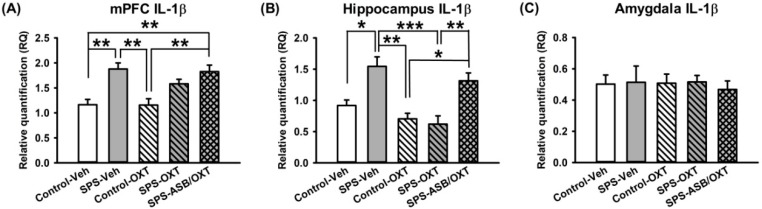
SPS and drugs (OXT and ASB) changed the mRNA expression of central *IL-1β*. Medial prefrontal cortex (mPFC) *IL-1β* levels were increased following SPS, which could not be reversed by OXT (**A**). OXT reversed SPS-elevated *IL-1β* levels in hippocampus, and ASB further blocked the OXT effect (**B**). SPS and drugs had no effect on amygdala *IL-1β* levels (**C**). *n* = 5 for each group; SPS = single prolonged stress, Veh = vehicle, OXT = oxytocin, ASB = atosiban, OXTR = oxytocin receptor. Bars represent mean ± SEM; * *p* < 0.05, ** *p* < 0.01, *** *p* < 0.001.

After the behavioral experiments, the rats were sacrificed and their brains were removed to measure mRNA levels of neuroinflammation factors (i.e., *IL-1β*, *IFN-γ*, and *TNF-α*) in the mPFC, hippocampus, and amygdala. Regarding mPFC *IL-1β* mRNA levels, our data revealed a significant difference among all groups (F_(4,20)_ = 9.002, *p* < 0.001) in which *IL-1β* mRNA levels were higher in the SPS-Veh group than in the control group either with OXT treatment (*p* = 0.002) or without it (*p* = 0.003), and *IL-1β* mRNA levels were also higher in the SPS-ASB/OXT group than in the control-Veh (*p* = 0.005) and control-OXT groups (*p* = 0.005) (Figure 3A). For hippocampal *IL-1β* mRNA levels, one-way ANOVA showed a significant difference among groups (F_(4,20)_ = 10.908, *p* < 0.001), deriving from the higher hippocampal *IL-1β* mRNA levels in the SPS-Veh group compared with the control-Veh (*p* = 0.011), control-OXT (*p* = 0.001), and SPS-OXT (*p* < 0.001) groups, and the hippocampal *IL-1β* mRNA levels were also increased in SPS-ASB/OXT group compared with control-OXT (*p* = 0.014) and SPS-OXT groups (*p* = 0.005) (Figure 3B). Finally, one-way ANOVA revealed that no significant difference of *IL-1β* level existed in amygdala among groups (Figure 3C).

**Figure 4:**
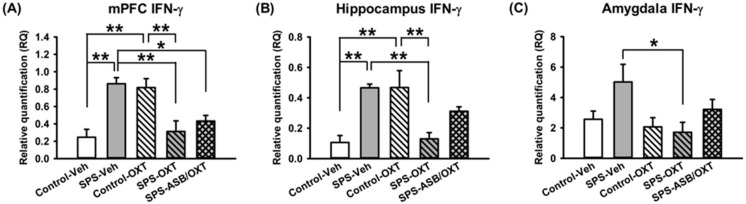
SPS changed the mRNA expression of central *IFN-γ* mRNA levels in a region-dependent manner, and OXT could reverse this phenomenon. *IFN-γ* levels in mPFC (medial prefrontal cortex) were increased following SPS, which could be reversed by OXT, and ASB was unable to block the OXT effect (**A**). Hippocampus *IFN-γ* levels were also increased following SPS, which could be reversed by OXT, and ASB further blocked the OXT effect (**B**). Amygdala *IFN-γ* levels seemed to be also increased following SPS, even though not significantly, which could be reversed by OXT, and ASB was unable to block the OXT effect (**C**). *n* = 5 for each group; SPS: single prolonged stress, Veh: vehicle, OXT: oxytocin, ASB: atosiban. Bars represent mean ± SEM; * *p* < 0.05, ** *p* < 0.01.

For mPFC *IFN-γ* mRNA levels, one-way ANOVA showed a significant difference among groups (F_(4,20)_ = 9.643, *p* < 0.001), further analysis revealed higher mPFC *IFN-γ* mRNA levels in the SPS rats compared with the control-Veh (*p* = 0.001), SPS-OXT (*p* = 0.004), and SPS-ASB/OXT rats (*p* = 0.027), in the other hand, the mPFC *IFN-γ* mRNA levels in the control-OXT rats were also higher than control-Veh (*p* = 0.002), and SPS-OXT rats (*p* = 0.008) (Figure 4A). Regarding hippocampal *IFN-γ* mRNA levels, a significant difference was found among all groups (F_(4,20)_ = 8.773, *p* < 0.001), further analysis revealed higher hippocampal *IFN-γ* mRNA levels in the SPS-Veh rats compared with the control-Veh (*p* = 0.003), and SPS-OXT rats (*p* = 0.005), and the hippocampal *IFN-γ* mRNA levels were also higher in the control-OXT rats compared with control-Veh (*p* = 0.003) and SPS-OXT (*p* = 0.005) rats (Figure 4B) significantly reversed the SPS-induced increment (F_(1,16)_ = 13.43, *p* = 0.005). Similar to mPFC and hippocampus, the data of amygdala *IFN-γ* mRNA levels in amygdala revealed a significant difference among groups (F(_4,20)_ = 2.993, *p* = 0.044), further analysis exhibited that OXT could reduce the amygdala *IFN-γ* mRNA levels under SPS (*p* = 0.04) (Figure 4C).

**Figure 5:**
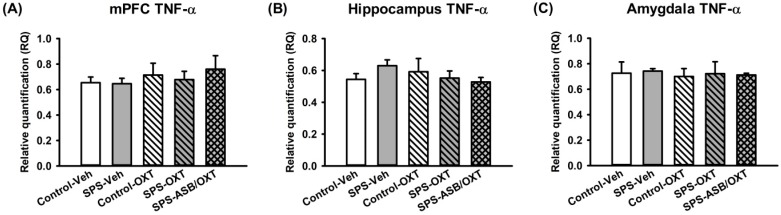
The SPS paradigm and drugs treatment had no effect on the *TNF-α* mRNA levels in the mPFC, hippocampus, or amygdala (**A**–**C**). *n* = 5 for each group; SPS: single prolonged stress, Veh: vehicle, OXT: oxytocin, ASB: atosiban. Bars represent mean ± SEM.

For the *TNF-α* mRNA levels, our data exhibited that the SPS and drugs (OXT and ASB) were no effects on *TNF-α* mRNA levels in mPFC, hippocampus, and amygdala (Figure 5A–C).

### 2.4. OXT Attenuated SPS-Induced Increment of Peripheral TNF-α and IL-1β Levels

**Figure 6:**
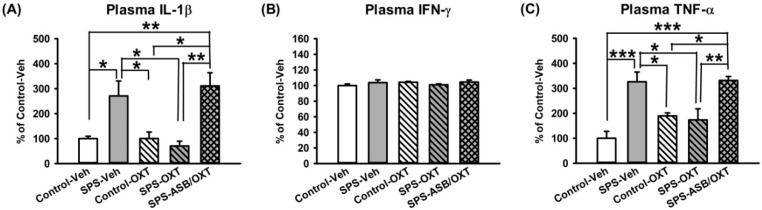
Peripheral IL-1β, IFN-γ, and TNF-α levels. OXT reversed SPS-induced increment of plasma IL-1β and TNF-α levels, and ASB seemed to block this effect with marginal significance (**A**,**C**). However, IFN-γ was shown to be insensitive to SPS, OXT, and ASB (**B**). *n* = 4 for each group; SPS: single prolonged stress, Veh: vehicle, OXT: oxytocin, ASB: atosiban. Bars represent mean ± SEM; * *p* < 0.05, ** *p* < 0.01, *** *p* < 0.001.

Peripheral IL-1β levels showed a significant difference among groups (F_(4,15)_ = 8.293, *p* = 0.001), further analysis revealed that SPS-Veh rats had higher plasma IL-1β levels than the control-Veh (*p* = 0.047), control-OXT (*p* = 0.048), and SPS-OXT rats (*p* = 0.017), SPS-ASB/OXT rats also had higher plasma IL-1β levels than the control-Veh (*p* = 0.012), control-OXT (*p* = 0.012), and SPS-OXT rats (*p* = 0.004) (Figure 6A). For peripheral IFN-γ levels, one-way ANOVA exhibited no significant difference among aleach groups (F_(4,15)_ = 0.81, *p* = 0.538) (Figure 6B). Finally, peripheral TNF-α levels showed a significant difference among all groups (F_(4,15)_ = 11.117, *p* < 0.001), deriving from higher plasma TNF-α levels in the SPS-Veh rats than in the control-Veh (*p* = 0.001), control-OXT (*p* = 0.042), and SPS-OXT rats (*p* = 0.021), SPS-ASB/OXT rats also had higher plasma IL-1β levels than the control-Veh (*p* = 0.001), control-OXT (*p* = 0.033), and SPS-OXT rats (*p* = 0.016) (Figure 6C).

## 3. Discussion

**Figure 7:**
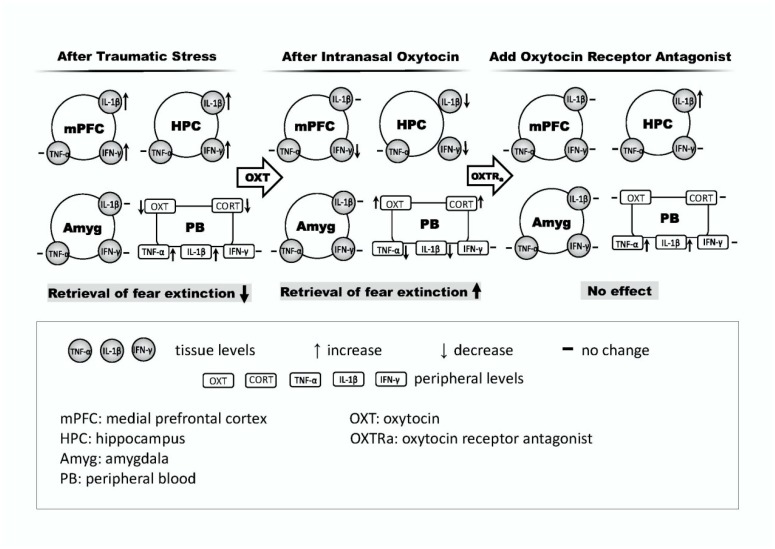
Hypothetical contributions of proinflammatory cytokines in fear circuit areas/peripheral blood on retrieval profiles of fear extinction after traumatic stress (**left panel**), intranasal oxytocin (**middle panel**), and oxytocin receptor antagonist (**right panel**).

In this study, we demonstrated that an SPS rodent model can be used to investigate the role of OXT in fear-related behaviors by exploring the effects of OXT on altered fear response and central and peripheral inflammation changes in rats who had previously experienced traumatic stress. We found that the SPS exposure exaggerated inflammation states in rats. In addition, we found that strengthening OXT signaling may reduce the levels of proinflammatory markers, which in turn would augment the efficacy of extinction training. These findings suggest the following: (i) SPS represents an ideal model for examining the effects of traumatic events on fear-related behavior and inflammatory changes; (ii) traumatic stress may impair both fear memory extinction ability and immune function; and (iii) OXT has potential therapeutic value for fear memory and immune compromise in PTSD (Figure 7). The interpretations of these findings are discussed below.

In line with previous evidence, the efficacy of SPS was validated behaviorally by an extinction failure of fear memory, as well as validated neurochemically by a lower plasma CORT level [7,31]. As both of these were sensitive to OXT manipulation, this supports the notion that hypocortisolemia reflects an excessive feedback in the HPA axis, which can be served as a biological hallmark observed in PTSD patients [34,35]. Please note that the similar response pattern to SPS of CORT and OXT (Figure 2) indicated that they both share the biological consequence following traumatic stress. It should be addressed that the CORT response to stress can be varied depending on the time period after stress; while acute stress increases the plasma CORT [36], after a longer time period after stress, such as in the condition of PTSD, the plasma CORT was found to be decreased [7,31]. Thus, our CORT findings, taken together with the finding of Wang et al. [36] that OXT reversed acute stress-increased plasma CORT, suggest that OXT may serve to normalize the CORT aberrations in both short-term and long-term biological responses to stress.

One of the aims of the present study was to examine the hypothesis of whether OXT signaling plays a role in the traumatic stress-induced fear memory abnormality [20]. Our findings that freezing behavior in the fear extinction test was significantly attenuated by SPS and then facilitated by intranasal OXT treatment (Figure 1) supports the hypothesis and is consistent with the findings of both human and animal studies that the fear extinction ability can be enhanced by augmenting OXT [37,38]. The extinction failure of SPS rats was associated a lower plasma OXT level, as the failure could be corrected by intranasal OXT treatment, implying a consistency of OXT levels from central to peripheral systems. Interestingly, the changes of plasma OXT were similar to the changes of plasma CORT, indicating that the effects of traumatic stress on fear-related freezing behavior were paired with the CORT and OXT plasma changes (Figure 2). Although no currently used laboratory methods have demonstrated a correlation between central and peripheral OXT in primates [39], Neumann and colleagues [40] demonstrated that increased brain and plasma OXT levels could be correlated after nasal and intraperitoneal OXT. Together with our findings, this suggests that the adjustment of OXT can contribute to an explanatory frameworks for potential therapeutics for PTSD.

For central *IL-1β* and *IFN-γ*, it was noted that SPS increased both of them in the mPFC and hippocampus. However, they responded differently to OXT administration. For *IL-1β*, OXT restored the SPS-decreased *IL-1β* in hippocampus but not mPFC. Please note that the stress-induced *IL-1β* effect was only found in the mPFC and hippocampus but not the amygdala, which is in accordance with previous evidence of fear learning [41] indicating that the amygdala is exempt from the stress-induced *IL-1β* effect. On the other hand, for *IFN-γ* in the mPFC and hippocampus, a diverse effect was noted according to rats’ trauma experience profiles, where OXT increased *IFN-γ* in control rats but decreased it in SPS rats (Figure 3 and Figure 4). Taken together, these findings may have implications in several aspects. First, increased levels of pro-inflammatory cytokines are considered a biological index of immune-associated stress [42], and here it appeared to sensitively reflect the therapeutic outcome of fear extinction abnormalities, as OXT diminished SPS-augmented *IL-1β* and *IFN-γ* in rats exhibiting higher freezing levels on the extinction retrieval day. Second, it is also possible that the elevation of pro-inflammation cytokines serves as a mechanism to mitigate the process of stress-associated reaction, as central IFN-γ is known to inhibit the release of adrenocorticotropic hormone (ACTH) [43]. Third, diverse effects of OXT on *IFN-γ* were found in the mPFC and hippocampus, suggesting a state-dependent influence of OXT. In other words, SPS-increased *IFN-γ* can be corrected by OXT; however, for rats without SPS, *IFN-γ* was raised by OXT.

It was noticed that although oxytocin exerted its effects on correcting the failure of fear extinction together with remedying central pro-inflammatory cytokines, the specificity of the effect was region-dependent, as it only exhibited a significant influence in the hippocampus where the effects on *IL-1β* were blocked by the OXT antagonist atosiban (Figure 3). This highlights the role of region-dependent inflammatory change in the pathoetiology of fear memory abnormality, in which hippocampus-dependent rather than cortex-dependent learning and memory is highly sensitive to the changes of pro-inflammatory cytokines [44,45]. This might be particularly relevant to our current paradigm to simulate a long-term stress reaction of PTSD, as IL-1β has been implicated in the process of fear memory formation and neuroplasticity [46] and hippocampal *IL-1β* mRNA expression and immunoreactivity were observed to increase in a time-dependent manner after stress [41].

As the distribution of OXT receptors differs across the brain, the central effects of OXT may be region-dependent [47,48]. Moreover, the brain regions sensitive to OXT have been implicated in learning and memory [48]. In the present study, the pro-inflammatory cytokines in the amygdala appeared to be different from those of mPFC and hippocampus. For example, *IL-1β* in the amygdala was unchanged following SPS and OXT manipulations, and *IFN-γ* did not exhibit the state-dependent, diverse effects of OXT as it did in the mPFC and hippocampus. It was noticed that the profiles of pro-inflammatory cytokines were also not consistent in rats following SPS and OXT manipulations. While *IFN-γ* was elevated in SPS rats, which could be reversed by intranasal OXT administration, *IL-1β* and *TNF-α* in the amygdala remained unchanged (Figure 3, Figure 4 and Figure 5). Note although the mPFC, hippocampus, and amygdala are the fear circuit areas of the brain, their biological performance can be different along with their anatomical/physiological profiles. For example, fear extinction functions to inhibit the expression of conditioned reaction via a possible top-down mechanism from the mPFC to the amygdala [49,50,51], thus the profiles of pro-inflammatory cytokines in the amygdala could be different from the others. As the OXT-mediated amygdala-associated fear reactivity is highly dependent on dose [37,52], it is possible that the dose of OXT employed in the present study was sufficient to regulate *IL-1β* and *TNF-α* in the mPFC and hippocampus, but not in the amygdala.

For peripheral cytokines, we demonstrated a similar response of IL-1β and TNF-α—the SPS-increased plasma levels of these two cytokines were reduced following the treatment of intranasal OXT, and these effects could be in turn noticeably blocked by the OXT antagonist atosiban, although they failed to reach statistical significance (New Figure 6). However, in contrast to the indifference of brain TNF-α levels in the mPFC, hippocampus, and amygdala, plasma TNF-α was found to be sensitive to SPS and OXT manipulations, in line with the high sensitivity profile of plasma TNF-α in human PTSD [53].

Several limitations/concerns of the present study should be addressed. First, similar to previous evidence that the peripheral profiles of cytokines appear to not necessarily be correspondent to their central profiles in the reaction to stress [54], the present study also found this inconsistency; while TNF-α became sensitive to SPS and OXT in the peripheral but not in the central profiles, the opposite was found for IFN-γ. The underlying mechanism of these changes remains unclear, possibly due to the sensitivity difference. In general, peripheral plasma is relatively non-specific, in contrast to the region-dependent brain level of a given cytokine. Second, due to the restrictive quota of the number of experimental rats, a group of non-SPS rats treated with ASB was not included in the present study. Thus, our data should be interpreted cautiously as ASB may have had some biological effects on the subjects [55]. Third, the effects of OXT and atosiban were determined based on a single-dose regimen; thus, some of the effects only reached marginal significance. A protocol employing multiple doses will be necessary in future studies.

In conclusion, we demonstrated that in the SPS model, OXT showed potential to treat many perspectives of PTSD. Behaviorally, it effectively amended the SPS-impaired fear extinction retrieval. Neurochemically, in a region-dependent manner, it reversed the SPS-increased pro-inflammatory cytokines. It is known that OXT has previously been employed in a clinical context to treat social interaction deficit [56]. The present study extends the potential of OXT in treating PTSD-associated neuroinflammation and fear memory abnormality.

## 4. Materials and Methods

### 4.1. Animals and PTSD Model

Male Sprague-Dawley rats (BioLASCO Taiwan Co., Ltd.) were used. The rats were aged 8 weeks and had been weaned upon arrival at the animal center of the National Defense Medical Center (Taipei, Taiwan). They were housed in groups of three in a temperature- and humidity-controlled holding facility with 12-h light/dark cycles (lights on from 07:00 to 19:00). Food (standard laboratory chow diet; Ralston Purina, St. Louis, MO, USA) and sterile water were available ad libitum. Rats were randomly assigned to control (control, *n* = 16, including 8 for vehicle and 8 for OXT) or SPS groups (*n* = 22, including 8 for vehicle, 8 for OXT, and 6 for OXT with OXT antagonist). The SPS procedure [27,28] consisted of the following steps. First, the rats were sequentially restrained in a plastic cone for 2 hours and then forced to swim in a tank of water (22-inch diameter, 20 °C) for 20 minutes. Following a 15-min recuperation period, they were exposed to diethyl ethyl vapor (Sigma, St. Louis, MO, USA) until they became anesthetized and unresponsive, and they were then immediately returned to their home cages and left undisturbed for 7 days, thus making them susceptible to impaired fear extinction retrieval [31,57]. During the SPS procedure, the control group rats remained in their home cages. All of the behavioral tests were conducted between 10:00 and 16:00, and all of the rats were tested at the same time every day when possible. The experimental procedures and ethics were approved by the National Defense Medical Center’s animal care committee (permission code: IACUC-15-113; permission date: 20 April 2015). The Laboratory Animal Center (LAC) of National Defense Medical Center granted a full accreditation from the Association for Assessment and Accreditation of Laboratory Animal Care International (AAALAC). All efforts were made to reduce the number of animals used and minimize their suffering during the experiments.

### 4.2. Experimental Design

Rats in SPS or control conditions were subjected to a pharmacological regime before behavioral testing of CDFC. CDFC was carried out 40 min after the intranasal administration of synthetic OXT (OXT 1 μg/μL, 2 × 10 μL; cat. no.: O4375, Sigma-Aldrich, St. Louis, MO, USA), which allowed OXT to reach its peak level in the brain [40,58], and the OXT receptor antagonist atosiban (ASB, cat. no.: A3480, Sigma-Aldrich) was administered intraperitoneally (5 mg/kg) 30 min prior to the delivery of the OXT [59]. Saline vehicle (Veh) was also intranasally administered to the rats not receiving OXT. The subgroups were thus as follows: control-Veh, control-OXT, SPS-Veh, SPS-OXT, and SPS-ASB/OXT. We administered a behavioral task to evaluate all the groups, namely the cue-dependent Pavlovian fear conditioning–extinction test (CDFC). Neurochemical data (plasma OXT and corticosterone (CORT) levels; cytokine interleukin 1β (IL-1β), TNF-α, and IFN-γ levels) were obtained by blood sampling taken from the apex of the rat heart under deep anesthesia to measure the peripheral and central effects of experiencing SPS.

### 4.3. Cue-Dependent Fear Conditioning-Extinction Test

This test was a 3-day paradigm to assess conditioned fear responses by measuring the percentage of freezing behavior in an operant chamber (30 × 24 × 26 cm^3^, plexiglass and aluminum; Clever Sys., Inc., Reston, VA, USA). An 8-W light-emitting diode (LED) bulb was positioned in the center of the wall to provide lighting (10 s, with an intertrial interval of 60 s) as a conditioned stimulus (CS), and 18 stainless steel rods (diameter = 5 mm, spaced 1.5 cm apart) composing the chamber floor delivered a foot shock (1 s, 1 mA) as the unconditioned stimulus (US). The chamber was housed in a wooden box to attenuate noise, and the wall of the wooden box contained a house light (75 W) and a fan (65 dB). The freezing behavior of the rats was monitored using a freezescan system (Clever Sys., Inc.). Two different contexts were used in this paradigm. In context A (day 1), a 1% acetic acid solution was placed in trays at the bottom of the chamber, with the house light and fan operating. In context B (days 2 and 3), the 1% acetic acid solution was replaced with a 1% ammonium hydroxide solution, a red light replaced the standard house light, and the fan was switched off. The rats were placed in the operant chamber without CS and US for 3 minutes prior to the first tone administration on each day. On day 1, the rats received five iterations of CS for habituation, and they subsequently received seven paired CS presentations that co-terminated with one US (conditioning stage). On day 2, the rats received 15 CS iterations (extinction stage). On day 3, the rats received one CS iteration (retrieval stage). The percentage of freezing time was recorded for each 70-s period (10-s CS and 60-s intertrial interval combined).

### 4.4. Plasma OXT and CORT Analyses

Blood samples were taken from the apex of the rat heart under deep anesthesia around 17:00 to 19:00 before scarification. Please note that in humans the largest difference of cortisol level presents in the time where the cortisol is in its highest level of the day (in the morning, after waking up) [60]. In rats, as they are nocturnal animals, their highest level of corticosterone presents around 17:00 to 19:00. Thus, we arranged the blood sampling to be performed during that time period. Blood samples were immediately collected using a mixture of 1 μL heparin sodium and differential centrifugation. The postnuclear supernatant was then aspirated and carefully transferred into a tube. The plasma OXT and CORT levels were assayed using an enzyme immunoassay (EIA) kit (no extraction required; No. 500440 and 501320, Cayman Chemical Company, Ann Arbor, MI, USA) with an absorbance of 407 nm, at which OXT levels in the brain extracellular fluid are directly proportional to OXT plasma levels [40,61].

### 4.5. Analysis of mRNA Expression

The rats were sacrificed through decapitation, and brain tissues containing the mPFC, hippocampus, and amygdala were rapidly dissected and immediately frozen at −70 °C. Tissues were homogenized in the lysis buffer of a MagNA Pure Compact RNA Isolation Kit using MagNA Lyser (Roche Molecular Diagnostic, Mannheim, Germany). Total RNA was extracted using the MagNA Pure Compact System (Roche). The quantity of RNA was determined using a NanoDrop One spectrophotometer (Thermo Scientific), and the DNA sequence was evaluated using Primer Express software. The primers were synthesized by Mission Biotech Ltd. (Taipei, Taiwan). Quantitative real-time polymerase chain reaction (qPCR) was used to analyze *IL-1β*, *IFN-γ*, and *TNF-α*. Sequences for the specific primer sets used in quantitative polymerase chain reaction (qPCR) were presented for each gene, as follows: *IFN-γ* (F: ATCGCCAAGTTCGAGGTGAA, R: TCCTTAGGCTAGATTCTGGTGACA), *IL-1β* (F: GCTGTGGCAGCTACCTATGTCTT, R: GTCACAGAGGACGGGCTCTTC), *TNF-α* (F: CAGACCCTCACACTCAGATCATCT, R: TCCGCTTGGTGGTTTGCTA). Additionally, *GAPDH* (F: GGTGGACCTCATGGCCTACA, R: CAGCAACTGAGGGCCTCTCT) was used as a housekeeping gene. RNA samples were reverse-transcribed for 120 min at 37 °C using a High-Capacity cDNA Reverse Transcription Kit (Applied Biosystems, Foster City, CA, USA), according to the supplier’s standard protocol. cDNA derived from 10 ng RNA was used for qPCR under the following conditions: 10 min at 95 °C, 40 cycles of 15 s at 95 °C, and 1 min at 60 °C using a 2× Power SYBR Green PCR Master Mix (Applied Biosystems) and 200 nM forward and reverse primers. Each assay was performed in triplicate on an Applied Biosystems 7900HT Real-Time PCR system, and expression fold changes were derived using the comparative C_T_ method, with *GAPDH* as an endogenous control and the control-Veh sample as a calibrator.

### 4.6. Plasma Cytokines Analyses

Samples of plasma (30 μL) were collected, and the three cytokines (IL-1β, IFN-γ, and TNF-α) were assayed using a cytometric bead array rat soluble protein master buffer kit (BD™ CBA Enhanced Sensitivity Flex Set System). Cytokine levels in the samples and standards were measured using a BD FACSCalibur™ Flow Cytometer to identify particles with fluorescence characteristics for both the bead and detector [62]. Enzyme-linked immunosorbent assay plates were read in a SPECTROstar Nano Microplate Reader (BMG LabTech) within 30 minutes of adding the stop solution. Optical density values were obtained and, after being blank-corrected, the mean ± SD was calculated for each sample, with absolute control group values of 100 for statistical analysis.

### 4.7. Data and Statistical Analysis

All statistical analyses were performed using SPSS (version 16.0, SPSS, Inc., Chicago, IL, USA) and SigmaPlot (version 12.1, Systat Software Inc., San Jose, CA, USA). The data of the fear conditioning test was analyzed by two-way of analysis of variance (ANOVA) with a between-subjects factor of “GROUP” and a within-subjects factor “TRIAL”. The data of the plasma endocrines levels (i.e., CORT and OXT) and central/plasma proinflammatory cytokines expressions were analyzed by one-way ANOVA with a between-subjects factor of “GROUP”. The significant main effects were then analyzed in *post hoc* comparisons using the Tukey method (for the consistent sample number comparison), Scheffe method (for the inconsistent sample number comparison). A threshold of *p* < 0.05 was considered statistically significant, and a threshold of *p* between 0.1 and 0.05 was considered statistically trend.
